# Tamoxifen induced hepatotoxicity via gut microbiota-mediated hyodeoxycholic acid depletion and Farnesoid X receptor signaling disruption

**DOI:** 10.1080/19490976.2025.2610077

**Published:** 2026-01-02

**Authors:** Yuchun Chen, Haiyan Du, Wenxin Zhou, Meirong Qin, Meifang Li, Yibao Jin, Yaning Xu, Chong Ma, Jiaxuan Xia, Yongshi Mo, Ning Chen, Houshuang Huang, Hao Li, Zhiyong Xie, Ping Wang, Yanjun Hong

**Affiliations:** a School of Pharmaceutical Sciences (Shenzhen), Sun Yat-sen University, Shenzhen, China; b Shenzhen Key Laboratory of Drug Quality Standard Research, Shenzhen Institute for Drug Control, Shenzhen, China; c Department of Pharmacology and Pharmacy, The University of Hong Kong, Hong Kong, China; d Department of Hepatology, Shenzhen Traditional Chinese Medicine Hospital, The Fourth Clinical Medical College of Guangzhou University of Chinese Medicine, Shenzhen, China

**Keywords:** Tamoxifen-induced liver injury, gut microbiota, hyodeoxycholic acid, Farnesoid X receptor signaling

## Abstract

Tamoxifen (TAM) is a widely used estrogen receptor modulator for breast cancer treatment. However, TAM exhibits significant hepatotoxicity in the clinic, affecting nearly 50% of patients and thereby limiting its clinical utility. The specific mechanisms underlying TAM-induced liver injury remain poorly understood. In this study, we elucidated the mechanistic role of the gut microbiota in the hepatotoxicity associated with TAM. TAM administration induced substantial liver injury and gut microbiota dysbiosis in mice, characterized by an increased abundance of *Escherichia* and a reduction in *Lachnospiraceae NK4A136 group*. These microbial shifts resulted in decreased levels of total fecal bile acids (BA), particularly hyodeoxycholic acid (HDCA), which was inversely correlated with TAM-induced liver injury. Additionally, TAM disrupted BA homeostasis by enhancing intestinal Farnesoid X receptor (FXR) activity and concurrently stimulating hepatic BA synthesis through an alternative nonintestinal FXR mechanism. Notably, gut microbiota depletion reversed these effects, demonstrating the critical role of the microbiota in modulating the gut‒liver FXR axis in TAM-induced liver injury. Fecal microbiota transplantation (FMT) further confirmed that TAM directly stimulated hepatic BA synthesis through a microbiota-dependent mechanism. The disruption of the gut‒liver BA‒FXR axis impaired enterohepatic BA circulation, contributing to the liver toxicity associated with TAM administration. Importantly, HDCA supplementation restored the gut‒liver BA‒FXR axis and alleviated TAM-induced liver injury. These findings highlight the intricate relationship between TAM, gut microbiota, and BA metabolism, suggesting that targeting the gut–liver FXR axis with HDCA may serve as a promising therapeutic strategy for alleviating TAM-associated liver injury.

## Graphical abstract

**Figure:**
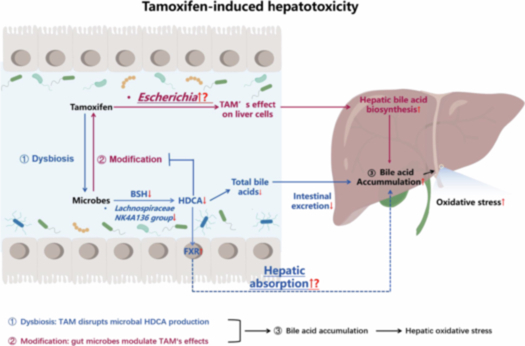

## Introduction

Breast cancer is the most prevalent malignant neoplasm globally, with estrogen receptor-positive breast cancer being the predominant subtype, accounting for nearly 70% of all cases.^1^ Tamoxifen (TAM), a selective estrogen receptor modulator, is widely used as a first-line treatment for breast cancer.^2^^,^^3^ However, TAM treatment has been associated with significant liver injury, affecting 30.4%–56.2% of patients in clinic.^4^^,^^5^ Although the hepatotoxicity induced by TAM has been linked to an increased generation of reactive oxygen species,^6^ the specific underlying mechanisms remain incompletely elucidated. Recently, emerging evidences demonstrated the critical role of drug-gut microbiota interactions in therapeutic outcomes.^7^^,^^8^ Drugs can modulate the composition of gut microbiota, while gut microbiota, in turn, can influence drug efficacy and toxicity.^7^^,^^8^ It is interesting to note that TAM, despite being a human-targeted drug, exhibits broad-spectrum bactericidal activity.^9^ Additionally, specific bacteria can modulate TAM-induced oxidative stress through influencing host fatty acid metabolism in *Caenorhabditis elegans* models.^10^ Given these potential interactions between TAM and gut microbiota, we hypothesized that gut microbiota may play a pivotal role in modulating TAM-associated liver injury.

The gut–liver axis refers to the bidirectional communications between the liver and the gut, involving interactions with gut microbes and modulated by genetic and environmental factors.^11^ Nutrients, bacterial products, and bile acids play crucial roles in regulating host metabolism and immune responses, which in turn shape the composition and function of the gut microbiota.^11^ Bacterial metabolites, particularly bile acids (BAs), serve as critical signaling molecules that influence the gut–liver axis, thereby regulating both gut and liver functions.^12^^,^^13^ Disruption of this host-microbe crosstalk has been implicated in the pathogenesis of various liver diseases, including drug-induced liver injury (DILI).^14^ BAs effectively regulate their own metabolism and transport via the gut–liver axis.^15^ The impairment of BA metabolism, transport, and signaling can lead to cholestatic liver injury,^16^ characterized by mitochondrial dysfunction and subsequent oxidative stress.^17^ Building on these findings, we hypothesized that TAM may disturb gut microbiota homeostasis and impair gut–liver axis function, ultimately leading to liver toxicity.

This study aims to elucidate the mechanistic role of gut microbiota in TAM-induced hepatotoxicity. First, we demonstrated that the absence of gut microbiota mitigated TAM-induced hepatic oxidative stress by using a pseudo germ-free mouse model. The results from 16S rDNA sequencing revealed that TAM significantly altered the composition of gut microbiota, characterized by a marked increase in the abundance of *Escherichia* and a decrease in *Lachnospiraceae NK4A136 group*. Importantly, these microbial shifts were significantly associated with the suppression of the secondary BA biosynthesis pathway. Fecal BA analysis further confirmed this robust connection in which TAM exposure led to a decrease in hyodeoxycholic acid (HDCA) levels, which in turn activated intestinal FXR signaling. Concurrently, the altered microbiota in turn modulated TAM's effect on liver cells that increased downstream hepatic BA production, which was validated by FMT experiments. These findings indicate that TAM disrupts the gut‒liver HDCA‒FXR axis, thereby leading to the dysregulated BA metabolism. Moreover, HDCA supplementation reversed these effects of TAM in mice, restoring the gut‒liver BA‒FXR axis and mitigating oxidative stress in the liver. Collectively, our study underscored the therapeutic potential of HDCA in alleviating TAM-associated liver toxicity.

## Materials and methods

### Animals

Female C57BL/6J mice (6–7 weeks old) were purchased from Guangdong Vital River Laboratory Animal Technology Co., Ltd. All animals were housed under 12-h light/dark cycle with ad libitum access to food and water. To minimize cage effects and normalize the gut microbiota composition, the mice were cohoused for 3 weeks prior to experimentation.^18^ Corncob bedding and drinking water were refreshed every 3–4 d. All experiments were conducted at the Shenzhen Institute for Drug Control in compliance with protocols approved by the Institutional Animal Care and Use Committee (202209082).

### Establishment of TAM-induced hepatotoxicity mouse model

The TAM-induced hepatotoxicity model (200 mg/kg) was established based on previously published studies with minor modifications.^19^ Briefly, TAM was dissolved in 0.5% sodium carboxymethyl cellulose (CMC-Na) by oral administration. The mice were randomly allocated into six groups (*n* = 5–10) and administered either vehicle control (0.5% CMC-Na) or TAM solution (200 mg/kg) once daily for 4, 8, or 16 weeks: 4w-control, 8w-control, 16w-control, 4w-TAM, 8w-TAM and 16w-TAM groups.

### Antibiotics treatment (ABX) experiment

The mice were randomly assigned to four groups (*n* = 9–10): WT-control (wild-type + vehicle), WT-TAM (wild-type + TAM), ABX-control (antibiotic-treated + vehicle), and ABX-TAM (antibiotic-treated + TAM) groups. To deplete the gut microbiota, ABX groups received a cocktail of broad-spectrum antibiotics (1 g/L neomycin, 1 g/L ampicillin, and 0.5 g/L vancomycin) in the drinking water and a daily oral gavage of 2 mg metronidazole.^20^ WT groups received normal drinking water throughout the study. After 3 d of antibiotic pretreatment, all mice began daily oral administration of either vehicle control (0.5% CMC-Na) or TAM solution (200 mg/kg) for 4 weeks (as shown in Figure 2A).

### Fecal microbiota transplantation (FMT)

FMT was performed over a 4-week period following published methods.^21^^,^^22^ The mice were randomly allocated into two groups (*n* = 10): FMT-control (recipients of microbiota from WT-control donors) and FMT-TAM (recipients of microbiota from WT-TAM donors) groups. Prior to FMT, recipient mice underwent gut microbiota depletion via daily oral gavage for five consecutive days with an antibiotic cocktail consisting of ampicillin (200 mg/kg), neomycin (200 mg/kg), metronidazole (200 mg/kg), and vancomycin (100 mg/kg). To verify the efficacy of antibiotic treatment in depleting the gut microbiota, total bacterial load was detected by quantitative PCR using Universal 16S primers (forward: 5′-TCCTACGGGAGGCAGCAGT-3′; reverse: 5′-GGACTACCAGGGTATCTAATCCTGTT-3′).^23^ For FMT preparation, fresh fecal samples collected from WT-control and WT-TAM donor mice were homogenized in PBS containing 20% glycerin (20 mg feces/mL) and centrifuged at 100 × *g* for 10 min at 4 °C. The resulting fecal supernatant was collected, aliquoted, and stored at −80 °C until use. The transplantation protocol consisted of an initial intensive phase with daily oral administration of 200 μL of fecal supernatant for three consecutive days, followed by a maintenance phase with administration every 2 d through the 4-week experiment period.

### Hyodeoxycholic acid (HDCA) and obeticholic acid (OCA) intervention study

Following 12 weeks of control or TAM treatment, the mice underwent an additional 4-week intervention period with the following experimental groups (*n* = 4–6): (1) Vehicle group: received continued control or TAM solution along with 0.5% CMC-Na vehicle; (2) OCA group: received control or TAM solution plus OCA (10 mg/kg/d in 0.5% CMC-Na); (3) HDCA/100 mg/kg group: received control or TAM solution plus HDCA (dissolved in 0.5% CMC-Na); (4) HDCA/150 mg/kg group: received control or TAM solution plus HDCA. All the solutions were administered daily by oral gavage. The dosages of OCA and HDCA were selected based on previously published studies.^24-26^

### Targeted inhibition of bacterial β-glucuronidase (GUS)

The mice were randomly assigned to four groups (*n* = 8): control-vehicle, control-GUS inhibitor (GUSi), TAM-vehicle, and TAM-GUSi. Following published protocols,^27^^,^^28^ the bacterial GUS inhibitor (UNC10201652, MCE) was dissolved in 100% DMSO (2 mg in 200 μL) and then diluted with water to 20 mL to reach a final concentration of 0.1 μg/μL. GUSi was administered for 4 weeks by oral gavage twice per day (every 10 h, 10 μg/d). The vehicle groups received an equivalent volume of 1% DMSO (100 μL twice per day).

### Biochemical assay

Following a 12-h overnight fasting, blood samples were collected and centrifuged at 1000 × *g* for 10 min to obtain serum. Serum alkaline phosphatase (ALP) activity was quantified using a commercial assay kit (Nanjing Jian Cheng Bioengineering Institute, Nanjing, China). For hepatic lipid profiling, liver tissue samples were analyzed to determine triglyceride (TG) and total cholesterol (TC) concentrations using standardized protocols (Nanjing Jian Cheng Bioengineering Institute, Nanjing, China). To evaluate the oxidative stress, liver tissues were homogenized in ice-cold buffer and centrifuged at 8000 × *g* for 10 min at 4 °C. The resulting supernatants were collected to determine the levels of reduced glutathione (GSH) and oxidized glutathione (GSSG) using commercial assay kits (Solarbio, Beijing, China) according to the manufacturer's instructions. The antioxidant activity was further evaluated by calculating the GSH/GSSG ratio.

### Tissue histopathology

Liver tissue samples were fixed in 4% paraformaldehyde and embedded in paraffin for hematoxylin and eosin staining. Slices were sectioned and stained with hematoxylin and eosin. Histological images were acquired using a Leica Aperio GT450 scanner. For immunofluorescence detection, paraffin-embedded intestinal tissues were sectioned at a thickness of 4 μm, deparaffinized in xylene, and rehydrated through a graded ethanol series. Antigen retrieval was performed by heating the sections in citrate buffer (pH 6.0) at 95 °C for 15–20 min using a microwave or water bath. After cooling to room temperature, the sections were washed with PBS, permeabilized with 0.3% Triton X-100 for 10–15 min, and blocked with 5% bovine serum albumin (BSA) for 1 h to reduce nonspecific binding. Primary antibody (FXR, with a dilution of 1:100, Abcam 129089) was applied, and incubated overnight at 4 °C in a humidified chamber. After washing, the sections were incubated with the appropriate fluorophore-conjugated secondary antibody for 1 h at room temperature in the dark. Nuclei were counterstained with DAPI for 5 min. The slices were mounted with anti-fade mounting medium and visualized using a fluorescence microscope. Images were captured from multiple randomly selected fields per sample. The fluorescence intensity was quantified using ImageJ software.

### 16S rDNA gene sequencing

Cecal contents were collected under sterile conditions and immediately frozen at −80 °C until analysis. 16S rDNA gene sequencing was performed by Shanghai Majorbio Bio-Pharm Technology Co. Ltd. following a previously published method.^25^ Briefly, microbial DNA was extracted using a fecal DNA isolation kit (QIAGEN) according to the manufacturer's instructions. The variable V3‒V4 regions of 16S rDNA genes were amplified using universal bacterial primers 338F (5′-ACTCCTACGGGAGGCAGCAG-3′) and 806R (5′-GGACTACHVGGGTWTCTAAT-3′). The amplification products were separated on 2.0% agarose gels and purified using the QIAquick PCR purification kit (QIAGEN). Microbial DNA was sequenced on the Illumina MiSeq platform, and adapter-ligated DNA fragments were further sequenced following standard protocols. The standard pipeline for 16S amplicon analysis involved clustering sequences within a 97% similarity threshold into operational taxonomic units. The raw sequence data were then processed using the Majorbio Cloud Platform.

### Bile acid (BA) analysis

Fecal samples (approximately 50 mg) were homogenized in 5 mL of ice-cold methanol containing 10 µL of internal standard (chlorpropamide, 10 μg/mL).^29^ After vortexing, the samples were centrifuged at 14,000 × *g* for 15 min at 4 °C. The supernatant was collected, vacuum-dried, and reconstituted in 500 μL of 50% methanol. The sample solution was then filtered through a 0.22 μm membrane prior to LC‒MS/MS analysis. The BA analysis was performed using a Nexera LC-30 system (Shimadzu) coupled with an AB SCIEX 4500 triple quadrupole mass spectrometer (MS). Chromatographic separation was achieved using an ACQUITY HSS T3 column (100 × 2.1 mm i.d., 1.8 μm, Waters) maintained at 40 °C with a flow rate of 0.3 mL/min. The mobile phase consisted of 20% acetonitrile (A) and 80% acetonitrile (B) both in 10 mM ammonium acetate aqueous solution. The elution gradient was set as follows: 0–1 min, 5%–8% B; 1–10 min, 8%–14% B; 10–11 min, 14%–25% B; 11–15 min, 25% B; 15–16 min, 25%–50% B; 16–19 min, 50% B; 19–22 min, 50%–95% B; 22–24 min, 95% B; 24–24.5 min, 95%–5% B; and 24.5–26 min, 5% B. The MS parameters were set as follows: ionization mode: negative electrospray ionization (ESI−); capillary voltage: 4.5 kV; gas pressures: GAS1 (50 psi), GAS2 (60 psi); source temperature: 550 °C; curtain gas: 30 psi; and detection mode: multiple reaction monitoring (MRM).

### Bile salt hydrolase (BSH) activity assay

Fresh fecal samples (55 ± 5 mg) were homogenized in 250 μL of ice-cold PBS (pH 7.4) by vortexing for 2 min. Bacterial cells were lysed through sonication for 90 s with a 30 s interval in an ice bath. The lysates were then centrifuged at 15,000 × rpm for 30 min at 4 °C, and the resulting supernatants were collected for further analysis. The total protein concentration was determined using a Multiskan Sky microplate spectrophotometer (Thermo Scientific). The samples were diluted to 1 mg/mL in PBS for subsequent assays. BSH activities were assessed by measuring the generation of d4-CA from d4-TCA. The enzymatic reaction was conducted in 200 μL of sodium acetate buffer (3 mM, pH 5.2) containing 50 μM d4-TCA as the substrate and 0.1 mg/mL protein working solution, following established protocols.^30^ After incubation at 37 °C for 20 min, the reaction was immediately terminated by fresh-freezing in dry ice. The sample was mixed with 100 μL of methanol and centrifuged at 15,000 rpm for 20 min at 4 °C. The supernatants were collected to determine the BSH activity. The conversion of d4-TCA to d4-CA was quantified using the LC‒MS/MS method as described above.

### Quantitative real-time PCR

Total RNA was isolated from tissue samples using the traditional TRIzol method and reverse transcribed with the Hifair Ⅱ 1 st Strand cDNA Synthesis SuperMix for qPCR (gDNA digester plus). Real-time PCR was performed using Hieff qPCR SYBR Green Master Mix (High Rox Plus)^25^ on an Applied Biosystems QuantStudio 5 Real-Time PCR System. The real-time PCR primer sequences are provided in Table 1.

**Table 1.** Primer sequencing.

Gene names	Primers
*Gapdh*	F: 5′-TTGATGGCAACAATCTCCAC-3′
	R: 5′-CGTCCCGTAGACAAAATGGT-3′
*Fxr*	F: 5′-TGGGCTCCGAATCCTCTTAGA-3′
	R: 5′-TGGTCCTCAAATAAGATCCTTGG-3′
*Cyp7a1*	F: 5′-AACAACCTGCCAGTACTAGATAGC-3′
	R: 5′-GTGTAGAGTGAAGTCCTCCTTAGC-3′
*Cyp8b1*	F: 5′-CTAGGGCCTAAAGGTTCGAGT-3′
	R: 5′-GTAGCCGAATAAGCTCAGGAAG-3′
*Cyp27a1*	F: 5′-CCAGGCACAGGAGAGTACG-3′
	R: 5′-GGGCAAGTGCAGCACATAG-3′
*Cyp7b1*	F: 5′-GGAGCCACGACCCTAGATG-3′
	R: 5′-TGCCAAGATAAGGAAGCCAAC-3′
*β* **-*actin*	F: 5′-CTAAGGCCAACCGTGAAAAG-3′
	R: 5′-GCCTGGATGGCTACGTACA-3′
*Fgf15*	F: 5′-GCCATCAAGGACGTCAGCA-3′
	R: 5′-CTTCCTCCGAGTAGCGAATCAG-3′

### Statistical analysis

All the quantitative data are presented as mean ± standard error of the mean (SEM). Statistical analysis was conducted using the Statistical Package for the Social Sciences software (IBM, version 27). Independent samples t-test and one-way analysis of variance were performed for group comparisons. Statistical significance was defined at three levels: *p* < 0.05, *p* < 0.01, and *p* < 0.001. Data visualization was performed using GraphPad Prism software (version 9).

## Results

### TAM treatment induced liver injury in mice

**Figure 1.**
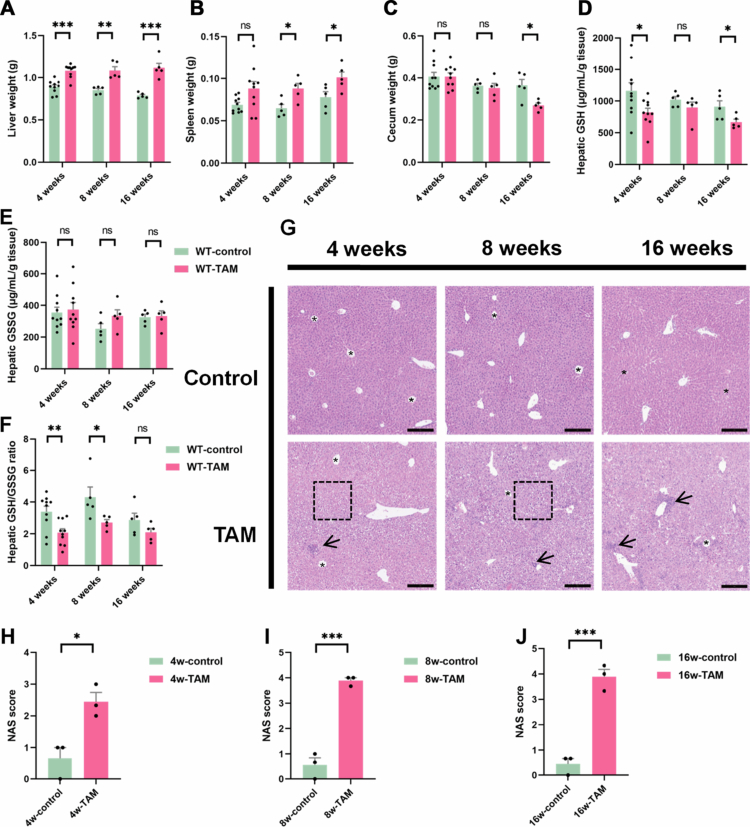
TAM induced hepatic oxidative stress in mice. (A) Liver weight, (B) spleen weight, and (C) cecum weight of mice undergoing 4-week (*n* = 10), 8-week (*n* = 5), or 16-week (*n* = 5) TAM or control treatment, respectively. (D) Hepatic GSH, (E) GSSG, and (F) the ratio. (G) HE staining of liver tissues. In (G), the dashed boxes and arrows indicate the histological changes of hepatic vacuolation and inflammation, while the asterisks (*) denote the structure of hepatic central veins. (H, I, and J) NAS score. Data were presented as the mean ± SEM. *, **, or *** indicated *p *< 0.05, 0.01, or 0.001 representing the comparisons between control and TAM-treated groups, respectively. The symbol of ‘ns’ represented no significant difference.

TAM administration (4, 8, and 16 weeks) induced significant liver injury in mice. Compared to the control group, TAM-treated mice displayed significantly increased liver weight (Figure 1A) and histopathological changes characterized by disorganized hepatic cords, pronounced liver cell vacuolation, and substantial inflammatory cell infiltration (Figure 1G–J). Additionally, we also found that TAM treatment increased the weight of spleen and cecal tissues (Figure 1B,C). Oxidative stress has been established as a critical mechanism underlying TAM-mediated liver toxicity.^6^ TAM administration significantly decreased hepatic GSH levels (Figure 1D), along with a markedly reduced GSH/GSSG ratio (Figure 1E,F). Notably, as shown in Figure 1F, TAM treatment for 4 and 8 weeks significantly reduced the hepatic GSH-to-GSSG ratio compared with that in control mice, indicating an early oxidative imbalance. Although mice subjected to 16 weeks of TAM treatment also exhibited a decrease in this ratio, the difference was not statistically significant compared to the control group (Figure 1F). These findings suggest that short-term TAM exposure induces oxidative stress in the liver, whereas prolonged exposure may trigger adaptive antioxidant responses that partially restore the GSH pool and redox equilibrium.

### TAM induced hepatotoxicity in a gut microbiota-dependent manner

**Figure 2.**
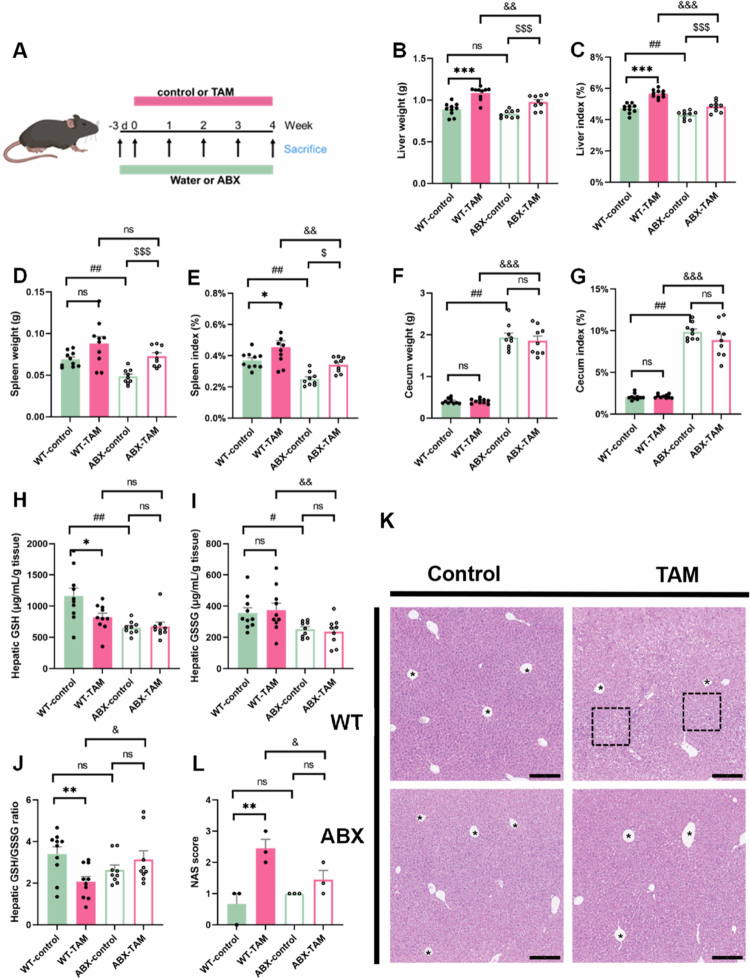
Depletion of the gut microbiota alleviated hepatotoxicity induced by TAM. (A) Intestinal bacteria depletion model. (B) Liver weight, (C) liver index, (D) spleen weight, (E) spleen index, (F) cecum weight, and (G) cecum index of WT (*n* = 10) and ABX (*n* = 9) mice with 4-week TAM or control treatment, respectively. (H) Hepatic GSH, (I) GSSG, and (J) GSH/GSSG ratio. (K) HE staining of liver tissues. In (K), the dashed boxes indicate the histological changes of hepatic vacuolation, while the asterisks (*) denote the structure of the hepatic central veins. (L) NAS score. Data were presented as the mean ± SEM. *, **, or *** indicated *p < *0.05, 0.01, or 0.001 representing the comparisons between WT-control and WT-TAM, respectively. ^#^ or ^##^ indicated *p < *0.05 or *p < *0.01 representing the comparisons between WT-control and ABX-control, respectively. ^$^ or ^$$$^ indicated *p < *0.05 or *p < *0.001 representing the comparisons between ABX-control and ABX-TAM, respectively. ^&^^,^^&&^, or ^&&&^ indicated *p < *0.05, 0.01, or 0.001 representing the comparisons between WT-TAM and ABX-TAM, respectively. The symbol of ‘ns’ represented no significant difference.

Antibiotic treatment was performed to explore the role of gut microbiota in TAM-mediated liver injury (Figure 2A). Using a broad-spectrum antibiotic cocktail to eliminate the intestinal microbiota, we observed substantial mitigation of TAM-induced liver damage, as demonstrated by significant reductions in both liver weight (Figure 2B) and the liver index (Figure 2C). The spleen weight and spleen index induced by TAM were also reduced through ABX treatment, along with a reduction on cecal tissues (Figure 2D–G). Histopathological results indicated marked improvement in hepatic architecture with antibiotic treatment, as evidenced by reduced hepatocyte swelling and rounding (Figure 2K,L). Importantly, ABX intervention modulated hepatic oxidative stress markers in TAM-treated mice, causing concurrent decreases in both GSH levels (Figure 2H) and GSSG production (Figure 2I), leading to a reduced GSH-to-GSSG ratio (Figure 2J). These findings suggest that gut microbiota depletion alleviates TAM-induced oxidative stress and hepatotoxicity, highlighting the critical role of intestinal bacteria in TAM-associated liver injury.

### Microbial β-glucuronidases are not a central driver of TAM-induced liver injury

The gut microbiota, often referred to as a “second liver” due to its extensive metabolic capacity, plays a pivotal role in modulating drug metabolism and toxicity.^31^ Within the intestinal lumen, microbial β-glucuronidase (GUS) enzymes can hydrolyze glucuronide conjugates, releasing the parent compounds and thereby facilitating their enterohepatic recirculation.^32^ This process is a well-established driver of drug toxicity—for example, GUS-catalyzed reactivation of SN-38, the active metabolite of irinotecan, induces severe gastrointestinal toxicity.^27^ TAM and its active metabolites are primarily inactivated through hepatic glucuronidation and subsequently excreted into the gut.^33^ Prior *in vitro* studies have demonstrated that TAM-glucuronide is a substrate for microbial GUS enzymes,^34^ implying that microbial GUS activity may modulate TAM elimination and toxicity.^33^^,^^34^

To test this hypothesis, we examined whether pharmacological inhibition of microbial GUS enzymes could mitigate TAM-associated hepatotoxicity. In TAM-treated mice, we measured fecal GUS activity and evaluated the potential protective effect of a GUS inhibitor (Supplementary Figure 3C). Surprisingly, TAM administration did not significantly affect fecal GUS activity after 4 or 8 weeks of treatment, whereas a notable reduction was observed after 16 weeks of TAM exposure (Supplementary Figure 3B). Following gut microbial ablation, fecal GUS activity was significantly decreased compared with the wild-type groups (Supplementary Figure 3A). Additionally, we assessed fecal GUS activity in FMT recipients, but no significant differences were detected between the FMT-control and FMT-TAM groups (Supplementary Figure 3I). Moreover, 4 weeks of GUS-inhibitor treatment failed to mitigate TAM-induced liver damage, as evidenced by unchanged liver weight gain, liver index, serum ALP levels, and liver vacuolation (Supplementary Figure 3D–H). Collectively, these findings suggest that microbial GUS is unlikely to be the primary driver of TAM-induced hepatotoxicity, highlighting the need to investigate additional microbial factors.

### TAM induced gut microbiota dysbiosis in mice

**Figure 3.**
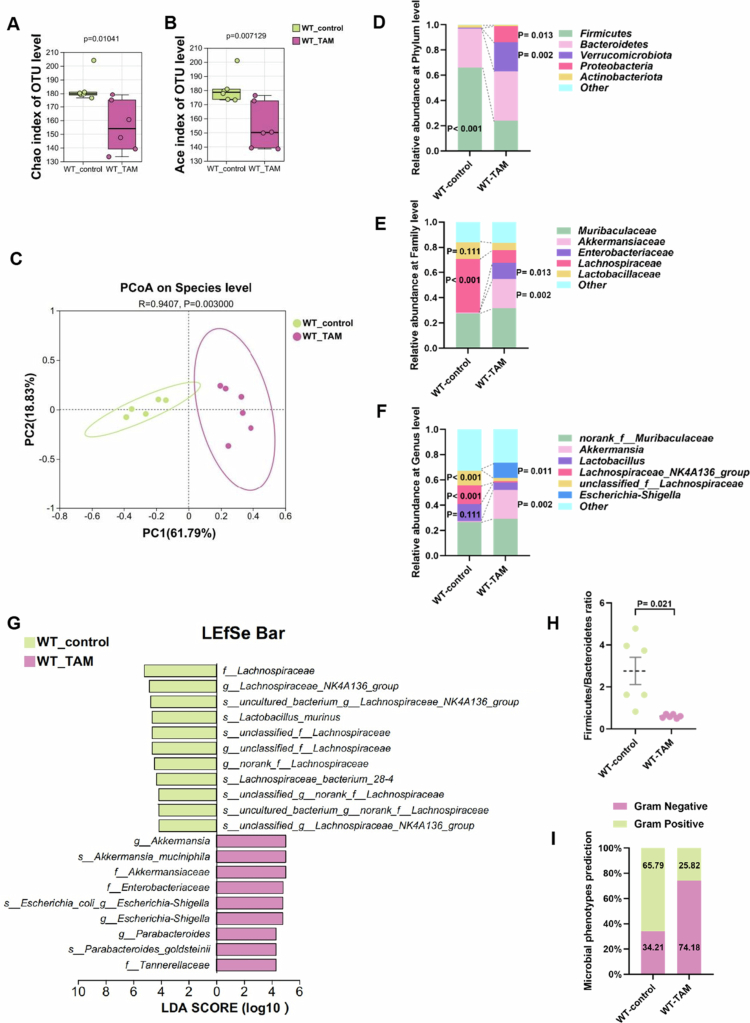
TAM remodeled the composition of the gut microbiota. (A) Chao index and (B) ace index of gut microbiota alpha-diversity in mice with 4-week TAM or control treatment (*n* = 6); OTU: operational taxonomic units. (C) PCoA of gut microbiota beta-diversity. Gut microbial composition at the (D) phylum, (E) family, and (F) genus level, respectively. (G) Linear discriminant analysis effect size (LEfSe) analysis of gut microbiota. (H) The ratio of *Firmicutes* and *Bacteroidetes*. (I) The proportions of Gram-positive and Gram-negative bacteria in the microbial community. Data were presented as the mean ± SEM.

To identify additional microbial drivers in TAM-induced hepatotoxicity, 16S rDNA sequencing was performed to comprehensively characterize TAM-induced alterations in gut microbiota composition. TAM treatment significantly reduced alpha-diversity, as evidenced by reduced Chao and Ace indices (Figure 3A,B). Beta-diversity analysis through Principal Coordinates Analysis (PCoA) demonstrated differences in microbial composition between the TAM-treated and control groups (Figure 3C). TAM administration induced a significant shift in the gut microbiota composition, with a reduction in Gram-positive bacteria and an increase in Gram-negative bacteria compared to the control group (Figure 3I). At the phylum level, while *Firmicutes* and *Bacteroidetes* remained dominant (Figure 3D), TAM treatment specifically reduced *Firmicutes* abundance, resulting in a decrease in *Firmicutes*-to-*Bacteroidetes* ratio (Figure 3H), a well-established dysbiosis indicator. Notably, the relative abundance of *Lachnospiraceae* (a specific taxon belonging to *Firmicutes*) decreased across various taxonomic levels (Figure 3E–G). In contrast, the *Verrucomicrobiota* and *Proteobacteria* phyla significantly increased following TAM treatment (Figure 3D). At the family, genus, and species levels, the relative abundance of *Akkermansiaceae* and *Enterobacteriaceae* also showed a marked elevation (Figure 3E–G). These findings demonstrate that TAM exerts a significant impact on gut microbial composition.

### TAM downregulated the secondary bile acid biosynthesis pathway

**Figure 4.**
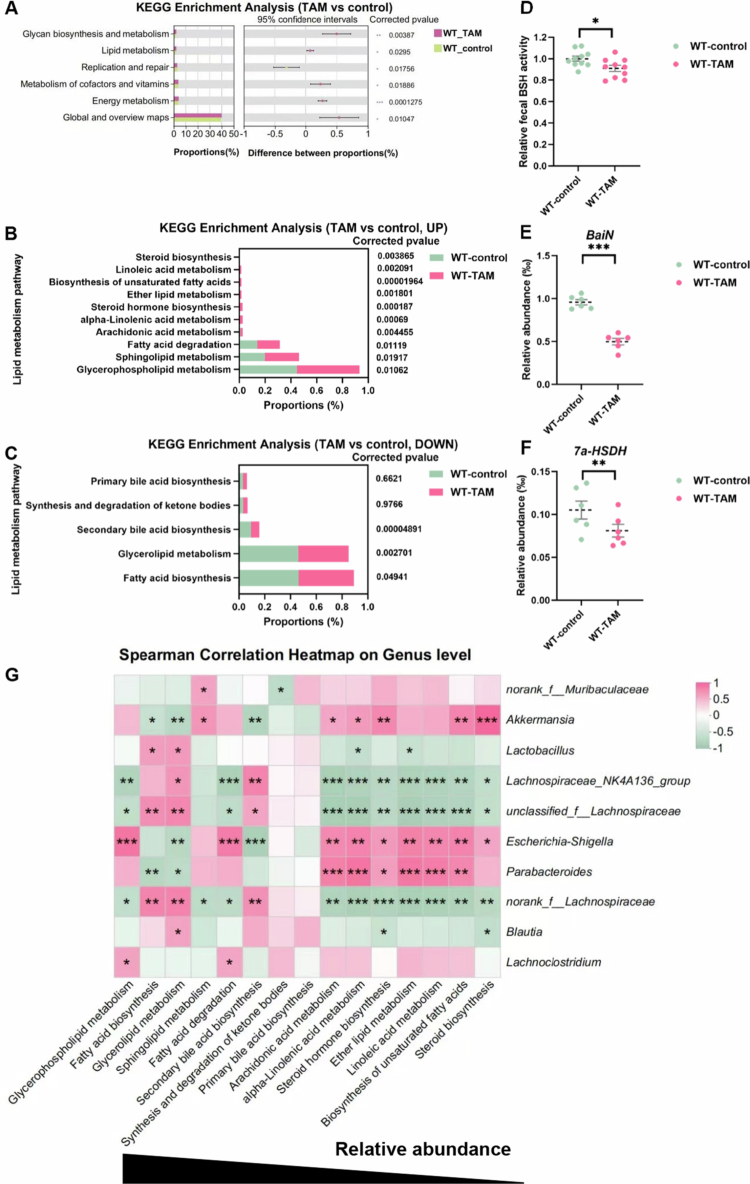
TAM reduced secondary BA biosynthesis signaling. (A) KEGG analysis of gut microbiota in mice with 4-week TAM or control treatment (*n* = 6). (B) Upregulated signaling of lipid metabolism in the TAM group. (C) Downregulated signaling of lipid metabolism in the TAM group. (D) Fecal BSH activity. (E) Relative abundance of BaiN. (F) Relative abundance of 7α-HSDH. (G) Spearman correlation analysis. Data were presented as the mean ± SEM. *, **, or *** indicated *p < *0.05, 0.01, or 0.001 representing the comparisons between control and TAM-treated groups, respectively.

KEGG pathway analysis was performed to investigate the functional consequences of TAM-induced gut microbiota dysbiosis (Figure 4A). While TAM treatment upregulated sphingolipid and glycerophospholipid metabolism (Figure 4B), it concurrently downregulated several critical pathways, including fatty acid biosynthesis, glycerolipid metabolism, and most notably, secondary bile acid (BA) biosynthesis (Figure 4C). Correlation analysis demonstrated a strong positive association between the secondary BA biosynthesis pathway and specific *Lachnospiraceae taxa* (*Lachnospiraceae_NK4A136_group*, *unclassified_f_Lachnospiraceae*, and *norank_f_Lachnospiraceae* genus) (Figure 4G), which are known to mediate secondary BA transformation through bile salt hydrolase (BSH)-dependent deconjugation.^35^ Importantly, TAM administration significantly inhibited fecal BSH activity (Figure 4D) and suppressed the gene expression of key enzymes in subsequent secondary BA conversion, including 3-dehydrobile acid delta 4,6-reductase (*BaiN*) (Figure 4E) and 7α-hydroxysteroid dehydrogenases (*7α-HSDHs*) (Figure 4F). Collectively, these findings demonstrate that TAM disrupts the secondary BA biosynthesis pathway, potentially attributed to the depletion of *Lachnospiraceae* genera.

### TAM disrupted microbial-dependent HDCA production

**Figure 5.**
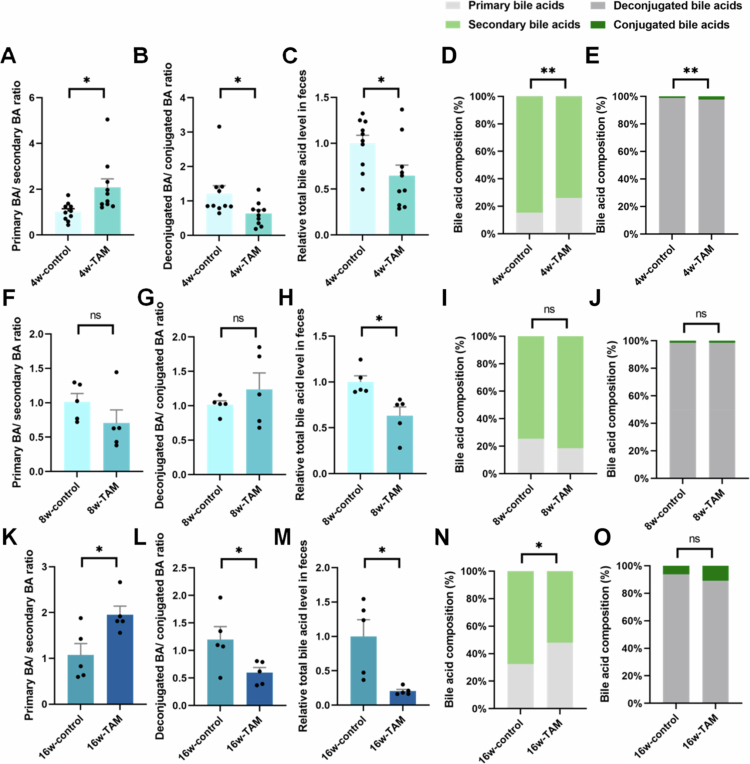
TAM reshaped the fecal BA profile and excretion. (A, F, and K) The ratio of primary and secondary BAs in mice undergoing 4-week (*n* = 10), 8-week (*n* = 5), or 16-week (*n* = 5) TAM or control treatment, respectively. (B, G, and L) The ratio of deconjugated and conjugated BAs, respectively. (C, H, and M) Relative levels of total BAs in fecal samples. (D, I, and N) The proportions of primary and secondary BAs. (E, J, and O) The proportions of deconjugated and conjugated BAs. Data were presented as the mean ± SEM. * or ** indicated *p < *0.05 or *p < *0.01 representing the comparisons between control and TAM-treated groups, respectively. The symbol of ‘ns’ represented no significant difference.

**Figure 6.**
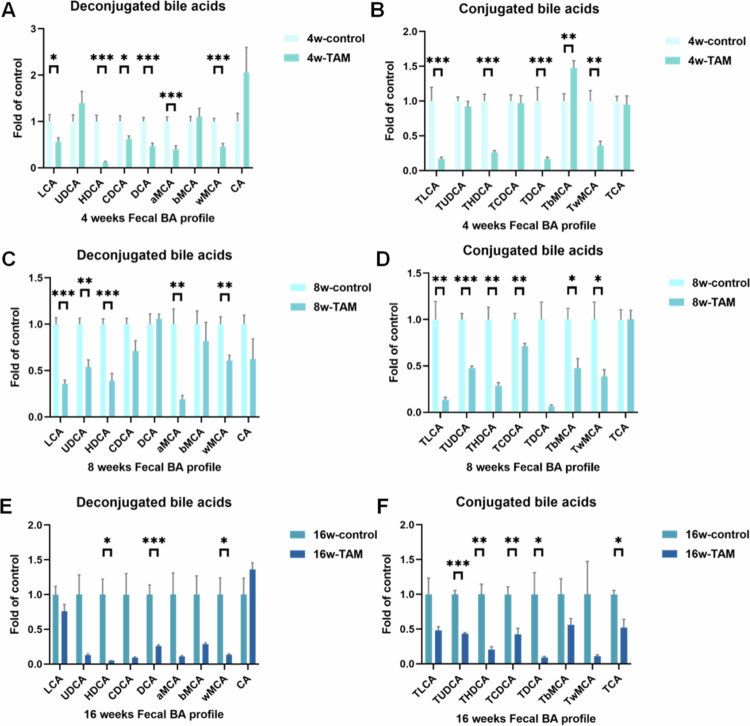
TAM significantly downregulated HDCA generation. (A, C, and E) The relative levels of deconjugated BAs in mice undergoing 4-week (*n* = 10), 8-week (*n* = 5), or 16-week (*n* = 5) TAM or control treatment, respectively. (B, D, and F) The relative levels of conjugated BAs. Data were presented as the mean ± SEM. *, **, or *** indicated *p < *0.05, 0.01, or 0.001 representing the comparisons between control and TAM-treated group, respectively. The absence of annotations in the chart indicates that there was no statistically significant difference between control and TAM group.

**Figure 7.**
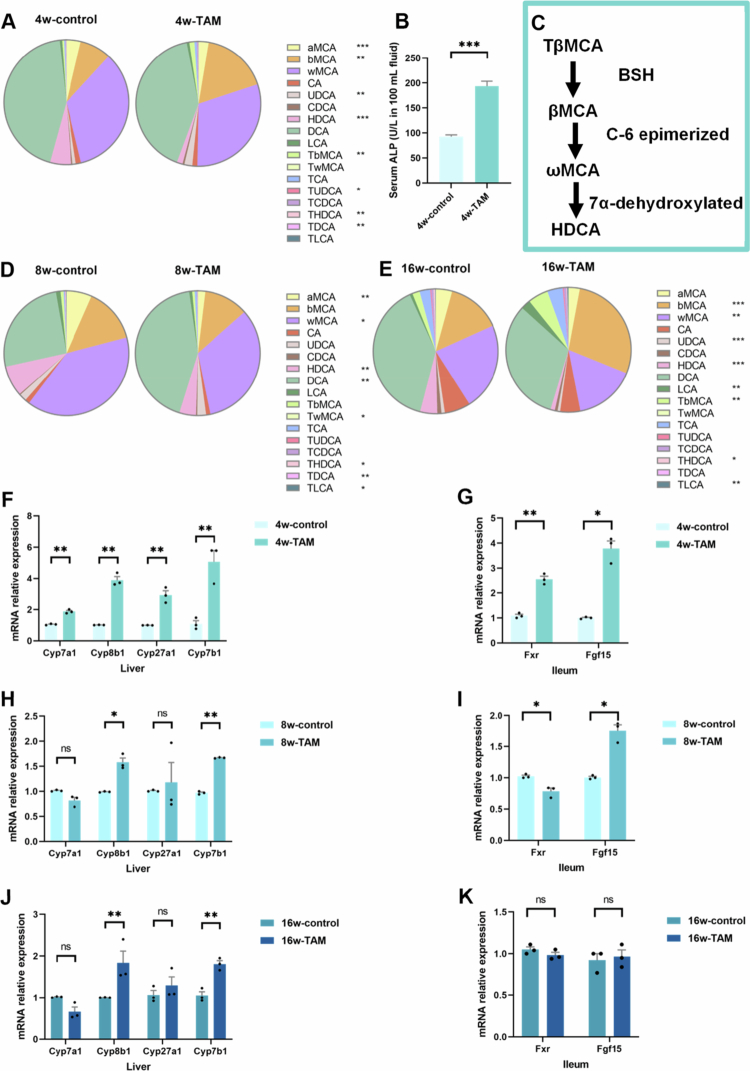
TAM disrupted the gut‒liver FXR axis. The composition of fecal BA profile in mice undergoing 4-week (A) (*n* = 10), 8-week (D) (*n* = 5), or 16-week (E) (*n* = 5) TAM or control treatment, respectively. (B) Serum ALP level. (C) Microbial conversion to generate HDCA. (F–K) The relative mRNA expression levels of gut–liver axis signaling genes. Data were presented as the mean ± SEM. *, **, or *** indicated *p < *0.05, 0.01, or 0.001 representing the comparisons between control and TAM-treated groups, respectively. The symbol of ‘ns’ represented no significantg difference.

The enterohepatic circulation efficiently maintains BA homeostasis by efficiently recycling BAs to the liver while excreting the remainder in feces.^36^ BA profiling revealed that TAM treatment significantly altered the fecal BA composition, with secondary BA levels markedly reduced (Figure 5A–O). Additionally, TAM induced significant decline of total BA concentrations in feces (Figure 5C,H,M, and Supplementary Figure 1C), which was closely associated with hepatic oxidative stress (Supplementary Figure 1D). In contrast, hepatic lipid profiles remained largely unchanged (Supplementary Figure 1A and B), except for a decline in total cholesterol levels after 16 weeks of exposure. Fecal BA analysis demonstrated a predominance of deconjugated BAs, including primary BAs (alpha-muricholic acid, αMCA; beta-muricholic acid, βMCA), and secondary BAs (deoxycholic acid, DCA; omega-muricholic acid, ωMCA; and hyodeoxycholic acid, HDCA) (Figure 7A,D,E). These deconjugated BAs are more hydrophobic, facilitating fecal excretion.^37^ TAM administration substantially decreased the deconjugated-to-conjugated BA ratio (Figure 5B,G,L), indicating reduced fecal BA excretion. During a 4-week period of administration, TAM significantly induced the depletion of ωMCA and HDCA (Figures 6A,B and 7A) while increasing βMCA and tauro-beta-muricholic acid (T-βMCA) levels. ωMCA is a key metabolite of βMCA generated through gut microbial C6-epimerization and can be metabolized to form HDCA via 7α-dehydroxylation,^38^ as depicted in Figure 7C. The inhibition of BSH activity by TAM likely impaired T-βMCA deconjugation to free βMCA, consequently reducing ωMCA and HDCA production (Figures 6A,B and 7A). Prolonged treatment with TAM (8 and 16 weeks) decreased T-βMCA and βMCA levels (Figure 6C–F), thereby limiting substrate availability for ωMCA and HDCA synthesis (Figure 7D,E). Importantly, correlation analysis established a strong positive association between HDCA levels and key hepatic antioxidant markers (GSH and GSH-to-GSSG ratio) (Supplementary Figure 1D). Furthermore, correlation analysis revealed significant associations between specific microbial taxa and BA profiles (Supplementary Figure 2B). Notably, *Lachnospiraceae_NK4A136_group* demonstrated a strong positive correlation with HDCA concentrations, whereas *Escherichia* showed a negative correlation with HDCA (Supplementary Figure 2B). These findings demonstrated that TAM significantly induces microbial HDCA depletion, which directly contributes to hepatic oxidative stress.

### TAM disturbed the gut–liver BA–FXR axis

**Figure 8.**
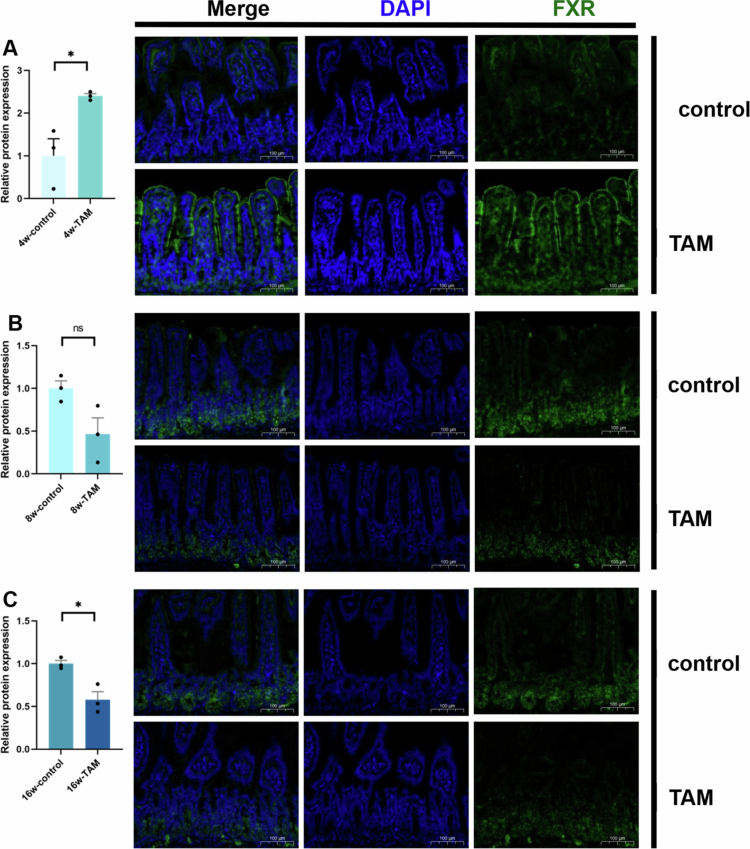
Long-term TAM treatment induced dynamic changes in intestinal FXR. Immunofluorescence (IF) detection of intestinal FXR in mice following 4-week (A), 8-week (B), and 16-week (C) of TAM treatment. * indicated *p < *0.05 representing the comparisons between control and TAM-treated groups. The symbol of ‘ns’ represented no significant difference.

BAs primarily exert their physiological effects by modulating FXR activity, which is abundantly expressed in both intestinal and hepatic tissues.^13^^,^^21^ As HDCA is a known inhibitor of intestinal FXR signaling that regulates hepatic BA synthesis,^24^^,^^39^ we investigated whether TAM-induced hepatotoxicity involves disruption of the gut–liver BA–FXR axis. During the initial 4 weeks of TAM administration, we observed significant intestinal FXR activation (Figure 7G). The activation of intestinal FXR has been linked to liver diseases through its role in increasing BA accumulation.^40^ After prolonged TAM treatment (8 and 16 weeks), the intestinal FXR signaling was decreased to control levels (Figure 7I and K). Consistently, the relative expression of intestinal FXR proteins demonstrated a significant increase at 4 weeks of TAM treatment, followed by a decline during prolonged administration (Figure 8). At 4 weeks, FXR activation may represent a compensatory response to early BA dysregulation and transient accumulation of primary BAs following TAM treatment. Given the progressively diminished total BA pool in the gut, the FXR signaling pathway initially normalizes and subsequently declines at later stages (8–16 weeks).

Interestingly, key enzymes governing hepatic BA synthesis (CYP7A1, CYP8B1, CYP27A1, and CYP7B1) were markedly upregulated (Figure 7F). This hyperactivation of hepatic BA synthesis persisted through longer treatment durations (8–16 weeks), with CYP8B1 and CYP7B1 remaining elevated (Figure 7H and J), indicating TAM's sustained impact on BA metabolism through compensatory activation of FXR-independent pathways. Despite enhanced hepatic BA production, fecal BA excretion decreased significantly, suggesting impaired enterohepatic BA circulation. This metabolic dysregulation was further confirmed by elevated ALP levels (Figure 7B, a clinical marker of cholestasis). Collectively, these results demonstrated that TAM-induced liver injury involves a disrupted gut‒liver axis characterized by complex microbiota‒BA‒FXR signaling.

### The disrupted gut–liver BA–FXR axis induced by TAM is microbial-dependent

**Figure 9.**
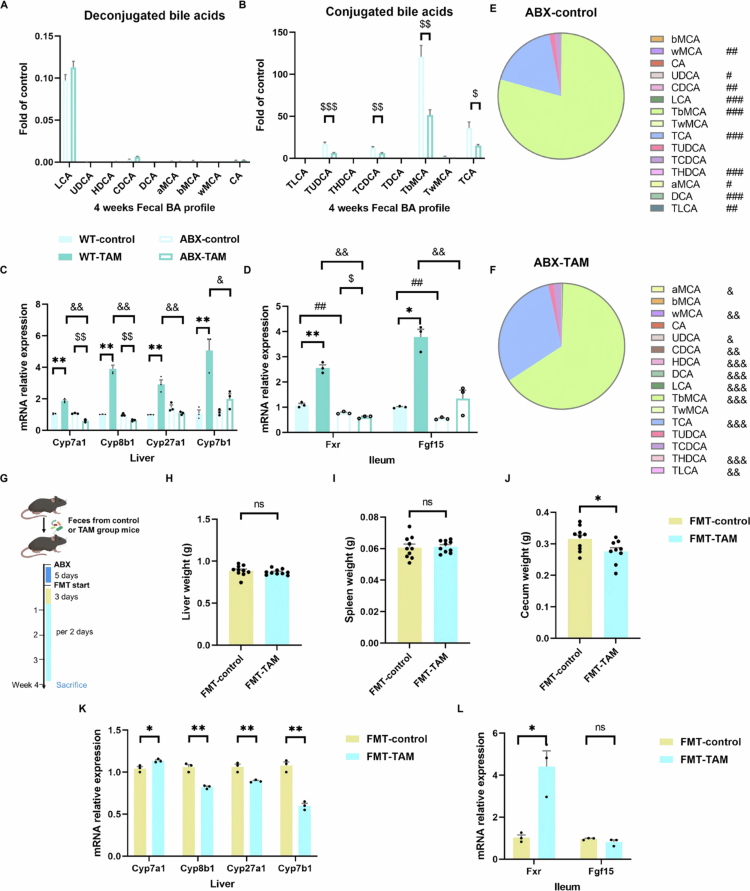
The gut microbiota mediated the gut‒liver BA‒FXR axis. (A) The relative levels of deconjugated BAs in ABX mice undergoing 4-week TAM or control treatment (*n* = 9). (B) The relative levels of conjugated BAs. (C and D) Relative mRNA expression levels of gut‒liver axis signaling genes. (E and F) Composition of the fecal BA profile. (G) FMT models. (H) Liver weight, (I) spleen weight, and (J) cecum weight in mice accepted with FMT (*n* = 10) from the control or TAM group, respectively. (K and L) Relative mRNA expression levels of gut‒liver axis signaling genes. Data were presented as the mean ± SEM. * or ** indicated *p < *0.05 or *p < *0.01 representing the comparisons between WT-control and WT-TAM, or FMT-control and FMT-TAM. ^#, ##^, or ^###^ indicated *p < *0.05, 0.01, or 0.001 representing the comparisons between WT-control and ABX-control, respectively. ^$, $$^, or ^$$$^ indicated *p < *0.05, 0.01, or 0.001 representing the comparisons between ABX-control and ABX-TAM, respectively. ^&, &&^, or ^&&&^ indicated *p < *0.05, 0.01, or 0.001 representing the comparisons between WT-TAM and ABX-TAM, respectively. The symbol of ‘ns’ represented no significant difference.

To investigate the role of the gut microbiota in the disrupted gut‒liver BA‒FXR axis induced by TAM, we conducted fecal BA profiling and examined FXR signaling pathway in ABX-treated mice. Our results demonstrated that ABX administration significantly elevated conjugated BA levels (Figure 9A,B), with a pronounced increase in T-βMCA in feces (Figure 9E,F). Given that T-βMCA is a well-established intestinal FXR antagonist, its accumulation suppresses fibroblast growth factor 15 (FGF15) expression, thereby stimulating hepatic BA synthesis. Notably, gut microbiota depletion attenuated intestinal FXR activation, particularly in TAM-treated mice (Figure 9D), likely due to the marked shift in fecal BA composition promoting T-βMCA accumulation (Figure 9E,F). Intriguingly, ABX treatment counteracted TAM-induced hepatic BA synthesis, as evidenced by the downregulation of key biosynthetic enzymes (CYP7A1, CYP8B1, CYP27A1, and CYP7B1) in the liver (Figure 9C). These findings suggest that the hepatic BA synthesis induced by TAM may be mediated by additional microbiota-dependent mechanisms.

To confirm the role of the gut microbiota in TAM-mediated disruption of the gut‒liver BA‒FXR axis, we further performed FMT from control and TAM-treated donors into antibiotic-pretreated recipient mice (Figure 9G). Prior to FMT, antibiotic treatment markedly decreased the total bacterial load in cecal samples (Supplementary Figure 4), demonstrating effective clearance of the majority of the gut microbiota. Strikingly, the gut microbiota from the TAM group recapitulated similar alterations in the recipient mice as those observed in the donor mice, including reduced cecum weight (Figure 9J) and enhanced intestinal FXR signaling activation (Figure 9L). Despite intestinal FXR activation, hepatic BA synthetic capacity was significantly reduced in FMT recipients, as evidenced by the downregulation of CYP8B1, CYP27A1, and CYP7B1; however, CYP7A1 expression was upregulated in the liver (Figure 9K). Therefore, TAM may directly act as a stimulatory signal on liver cells that enhances downstream BA production, a process influenced by the gut microbiota.

Overall, our findings demonstrated that the TAM-microbe interplay perturbs the gut‒liver axis through specific mechanistic pathways: on the one hand, TAM-induced gut microbiota dysbiosis reduces specific BA production (e.g. HDCA), potentiating intestinal FXR activation; on the other hand, the altered microbiota in turn modulates TAM's effect on liver cells that upregulates BA synthetic enzymes, indicating a nonintestinal FXR mechanism.

### HDCA ameliorated TAM-induced liver injury via the gut–liver BA–FXR axis

**Figure 10.**
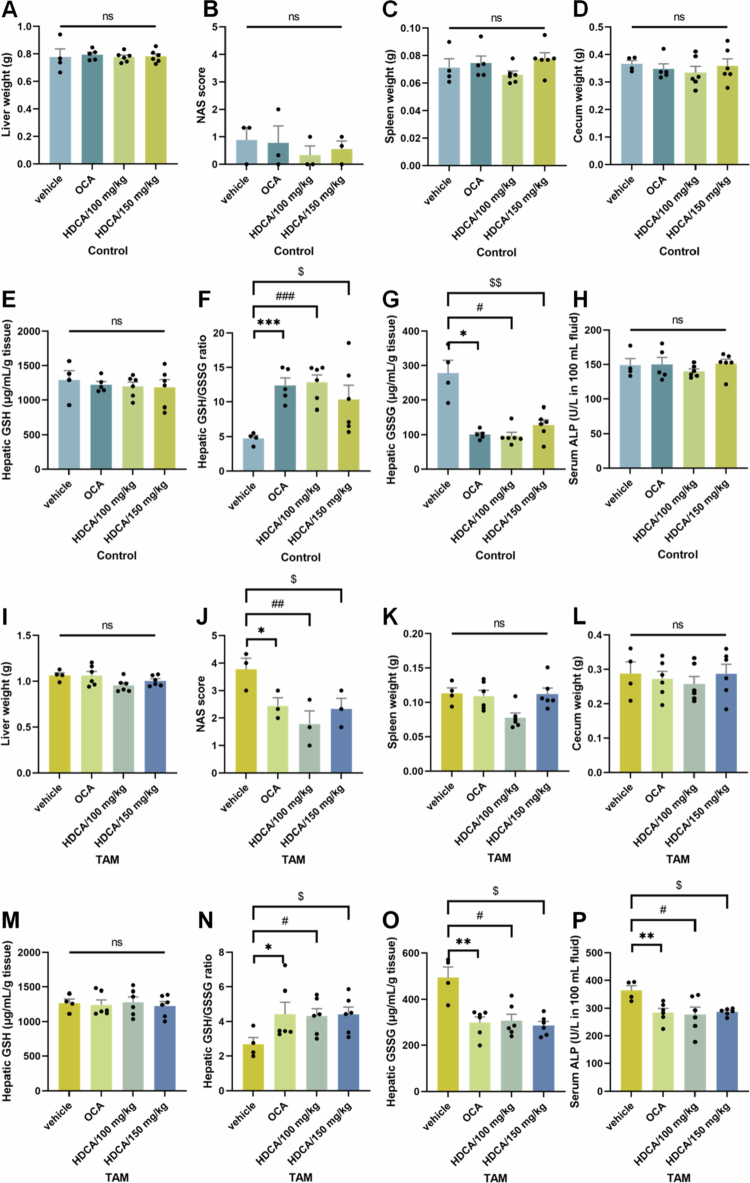
HDCA ameliorated liver injury induced by TAM. (A and I) Liver weight, (B and J) NAS score, (C and K) spleen weight, and (D and L) cecum weight of control or TAM (*n* = 4–6) mice undergoing vehicle, OCA, or HDCA treatment, respectively. (E and M) Hepatic GSH, (G and O) GSSG, and (F and N) the ratio. (H and P) Serum ALP levels. Data were presented as the mean ± SEM. *, **, or *** indicated *p < *0.05, 0.01, or 0.001 representing the comparisons between vehicle and OCA, respectively. ^#, ##^, or ^###^ indicated *p < *0.05, 0.01, or 0.001 representing the comparisons between vehicle and HDCA-100 mg/kg, respectively. ^$^ or ^$$^ indicated *p < *0.05 or *p < *0.01 representing the comparisons between vehicle and HDCA-150 mg/kg, respectively. The symbol of ‘ns’ represented no significant difference.

**Figure 11.**
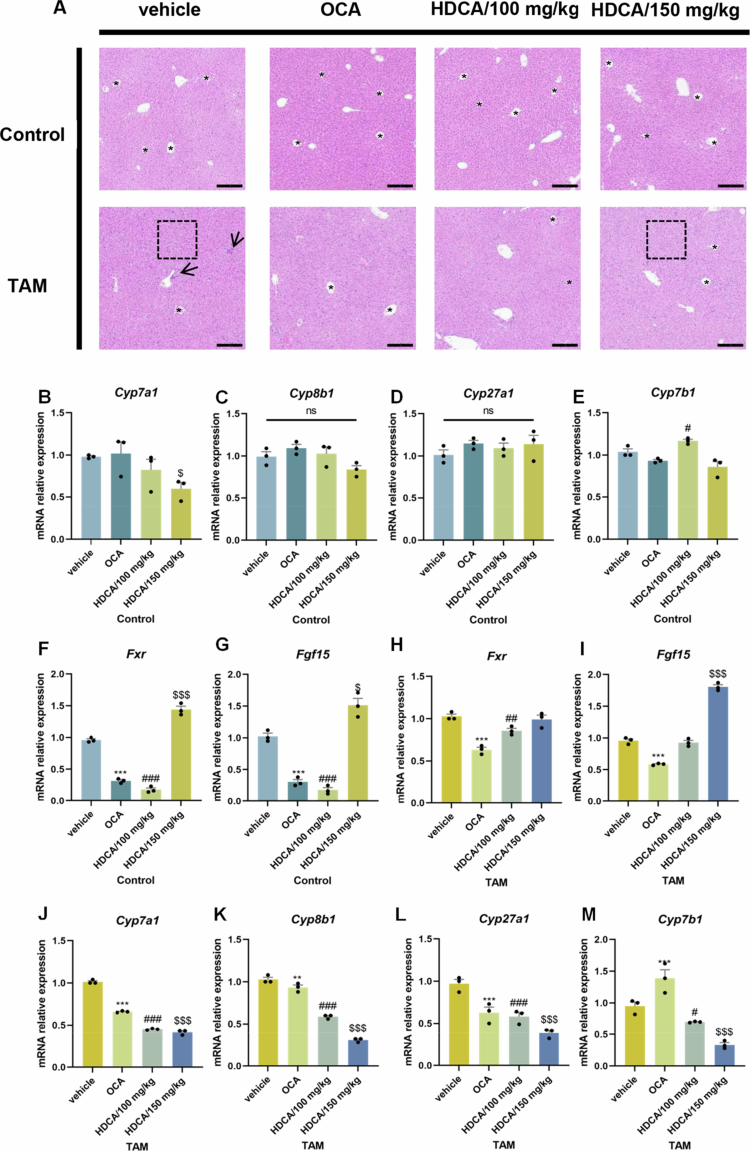
HDCA improved the disturbed gut–liver FXR axis induced by TAM. (A) HE staining of liver tissues in control or TAM (*n* = 4–6) mice undergoing vehicle, OCA, or HDCA treatment, respectively. (B–M) The relative mRNA expression levels of gut‒liver axis signaling genes. In Figure 10A, the dashed boxes and arrows indicate the histological changes of hepatic vacuolation and inflammation, while the asterisks (*) denote the structure of the hepatic central veins. Data were presented as the mean ± SEM. **, or *** indicated *p < *0.01, or *p < *0.001 representing the comparisons between vehicle and OCA, respectively. ^#, ##^, or ^###^ indicated *p < *0.05, 0.01, or 0.001 representing the comparisons between vehicle and HDCA-100 mg/kg, respectively. ^$^ or ^$$$^ indicated *p < *0.05 or *p < *0.001 representing the comparisons between vehicle and HDCA-150 mg/kg, respectively. The symbol of ‘ns’ represented no significant difference.

Emerging evidence highlights HDCA as a promising therapeutic agent for liver disorders through its regulatory effects on the gut‒liver FXR axis.^24^^,^^39^ Herein, the therapeutic potential of HDCA in TAM hepatotoxicity was further investigated. In the control group, HDCA treatment did not alter liver weight or morphology (Figures 10A,B and 11A), nor did it affect spleen or cecum weights (Figure 10C,D), indicating minimal physiological disturbance under basal conditions. Interestingly, HDCA significantly increased hepatic antioxidant activity, as evidenced by an elevated hepatic GSH/GSSG ratio (Figure 10E–G). Notably, in TAM-treated mice, HDCA exerted marked protective effects. Specifically, HDCA supplementation markedly alleviated oxidative stress and reduced serum ALP levels, improving BA overload in the liver (Figure 10M–P). Histopathological analysis confirmed that HDCA treatment mitigated morphological changes induced by TAM in the liver, reducing hepatic vacuolation (Figures 10J and 11A). These findings collectively demonstrated the protective effects of HDCA against TAM-induced liver injury.

We further evaluated the effects of HDCA on the FXR signaling pathway. HDCA exhibited conflicting roles in intestinal FXR signaling depending on the dosage administered. While HDCA acted as an antagonist at 100 mg/kg, it functioned as an agonist-like role at 150 mg/kg (Figure 11F–I), which is consistent with recent reports that high-dose HDCA induced cytotoxicity through FXR overactivation.^35^ Intriguingly, we found that both dosages of HDCA (100 and 150 mg/kg) restored TAM-disrupted hepatic BA synthesis by downregulation of key hepatic BA synthetic enzymes (CYP7A1, CYP8B1, CYP27A1, and CYP7B1) (Figure 11B–E, J–M). These findings suggested that the effects of HDCA in reducing hepatic BA overproduction potentially depend on additional regulatory mechanisms. It has been demonstrated that HDCA could promote the growth of bacteria associated with BA metabolism, specifically *Lachnospiraceae NK4A136 group.*^35^^,^^41^ This shift in microbial composition altered fecal BA pool, increasing total and secondary BAs while reducing primary BA levels.^35^ In contrast, following TAM administration, mice showed a significant enrichment of *Escherichia*, which was negatively correlated with both the protective *Lachnospiraceae NK4A136 group* (Supplementary Figure 2A) and the secondary BA biosynthesis pathway (Figure 4G). The hepatic BA overproduction induced by TAM may be mediated via the proliferation of specific bacteria, such as *Escherichia*, which could be inhibited by antibiotic or HDCA treatment. These findings uncovered that a 100 mg/kg dose of HDCA effectively protects against TAM-induced liver injury by suppressing intestinal FXR activation and regulating hepatic BA synthesis, likely via a microbiota-dependent manner.

OCA is a clinically approved semi-synthetic BA analog for the treatment of cholestatic liver diseases.^42^ In this study, OCA was employed to validate the critical role of the gut‒liver BA‒FXR axis in TAM-induced hepatotoxicity. Our results showed that OCA treatment significantly ameliorated TAM-induced liver injury (Figure 11A), as evidenced by reduced hepatic oxidative stress and serum ALP levels (Figure 10I–P). Moreover, OCA restored the gut‒liver BA‒FXR axis through the suppression of intestinal FXR activation and the downregulation of hepatic BA synthesis (Figure 11H‒M). These findings unveiled that improving the gut‒liver BA‒FXR axis promises therapeutic approaches for TAM-induced liver injury.

## Discussion

Drug-induced liver injuries are frequent and clinically significant adverse events that pose substantial medical challenges in patient care.^43^ Emerging evidence highlights the crucial role of the gut microbiota in modulating hepatotoxicity through both compositional and metabolic alterations.^43^ Characteristic gut microbiota alterations in drug-related liver toxicity typically manifest as decreased microbial diversity, reduced *Firmicutes* abundance, and elevated *Proteobacteria.*^44^ Our study revealed that TAM treatment recapitulated this microbial dysbiotic alteration in mice (Figure 3C,D). Specifically, TAM administration significantly depleted the beneficial *Lachnospiraceae NK4A136 group,* while enriching pathogenic *Escherichia* (Figure 3F,G). The resulting microbial dysbiosis significantly disrupted secondary BA biosynthesis pathways (Figure 4C and G). Specifically, TAM administration markedly suppressed microbial BSH activity (Figure 4D), thereby impairing the deconjugation of T-βMCA to free βMCA and subsequently reducing the production of HDCA (Figure 6A,C, and E). Our findings align with recent studies establishing the *Lachnospiraceae NK4A136 group* as a key microbial regulator of HDCA levels,^41^ positioning this bacterial taxon as a potential probiotic species for maintaining HDCA levels and mitigating TAM-associated liver injury.

Cholestasis serves as a key mechanism underlying drug-induced liver injury, characterized by impaired BA transport and metabolism. Then the hepatic accumulation of BAs during cholestasis triggers mitochondrial dysfunction and oxidative stress, driving disease pathogenesis.^17^ Furthermore, our findings demonstrate that TAM elicits parallel pathological changes through similar mechanisms, as evidenced by its disruption of BA metabolism and induction of oxidative stress (Supplementary Figure 1D). Additionally, the gut microbiota serves as a critical modulator of BA metabolism, exerting a profound influence on the pathogenesis and progression of cholestatic liver disorders.^45^ Furthermore, FXR not only serves as a master regulator of BA homeostasis but also is closely associated with the development of cholestasis in clinical settings, underscoring its significance as a key therapeutic target for cholestatic liver diseases.^45^ In our study, we demonstrated that TAM administration induces profound gut microbiota dysbiosis, which subsequently alters the intestinal BA composition. Most notably, TAM treatment significantly reduced HDCA levels, leading to consequent activation of intestinal FXR signaling (Figures 7G and 8A). Owing to the activation of FXR in the intestine, it can upregulate both intestinal bile acid-binding protein (I-BABP) and FGF15, which are key mediators of BA enterohepatic circulation.^40^ Increased I-BABP expression enhances transenterocytic BA transport, facilitating its entry into the portal circulation.^38^ Therefore, the inhibition of intestinal FXR underscores a potential therapeutic approach to mitigate pathological BA accumulation and facilitate clinical therapy of cholestatic liver diseases.^40^ We uncovered that HDCA supplementation can alleviate TAM-induced liver injury (Figure 10I–P). Mechanistically, our findings demonstrated that HDCA regulates BA metabolism through modulation of the gut‒liver BA‒FXR axis (Figure 11H‒M). Notably, we identified a dose-related opposite effect of HDCA on intestinal FXR signaling – while a dosage of 100 mg/kg exerted inhibitory effects, 150 mg/kg activated FXR (Figure 11F–I), which is consistent with recent reports of FXR-mediated intestinal cytotoxicity at elevated concentrations.^35^ Thus, it is recommended to use a 100 mg/kg dosage of HDCA to effectively inhibit intestinal FXR induced by TAM.

In addition, our findings revealed that TAM stimulates hepatic BA overproduction through a nonintestinal FXR-dependent mechanism (Figure 7F–K). The results from FMT experiment further indicate that TAM may exert a stimulatory effect on hepatic BA synthetic enzymes in a microbiota-dependent manner (Figure 9K,L). When given HDCA supplementation, it can downregulate the gene expression of hepatic enzymes involved in BA synthesis (Figure 11J–M). Extensive research has elucidated HDCA's critical role in maintaining BA homeostasis through two complementary mechanisms: 1) suppression of the intestinal FXR/FGF15 signaling pathway and 2) modulation of gut microbiota communities.^24^ HDCA has been reported to induce a favorable shift in fecal BA composition, characterized by reduced primary BAs and increased total and secondary BAs, potentially improving the enterohepatic circulation of BAs.^35^ Importantly, HDCA promotes the growth of BA-metabolizing bacteria, particularly the *Lachnospiraceae NK4A136 group*^35^—a key microbial taxon harboring BSH activity and capable of HDCA production.^41^ This emphasizes the opposing roles of TAM and HDCA in modulating the microbiota–BA axis. Specifically, TAM enhances hepatic BA synthesis through selective inhibition of BA-transforming bacterial taxa, whereas HDCA can counteract this effect.

Extensive preclinical studies have established HDCA as a promising therapeutic candidate for nonalcoholic fatty liver disease (NAFLD).^24^^,^^39^ Our study provided the first evidence that HDCA effectively attenuates TAM-induced liver injury in a mouse model (Figure 10I‒P), elucidating the pharmacological potential of endogenous HDCA in improving BA metabolism through the gut–liver microbiota–FXR signaling axis. This research trajectory may position HDCA as a novel adjunctive therapy for mitigating TAM-related hepatotoxicity while maintaining its anticancer efficacy. Future investigations should delineate the molecular mediators of HDCA's hepatoprotective effects, particularly its interactions with nuclear receptors beyond FXR, and evaluate its clinical translation potential in relevant patient populations.

We demonstrated for the first time that TAM treatment induces profound alterations in the gut microbial composition, characterized by selective enrichment of *Escherichia* and depletion of the *Lachnospiraceae NK4A136 group*, a key bacterial taxon involved in BA metabolism. These microbial shifts drive reductions in HDCA levels, thereby disrupting the gut‒liver BA‒FXR axis and exacerbating hepatic damage. Importantly, the 100 mg/kg dosage of HDCA effectively restored gut–liver BA–FXR signaling and mitigated TAM-induced liver injury in mice. Our findings revealed a complex intricate interplay between gut microbiota composition, HDCA signaling, and TAM-induced hepatotoxicity. These results underscore the therapeutic potential of HDCA for treating TAM-related liver disorder, offering a new approach for improving the safety profile of this widely used therapeutic agent.

### Limitation and prospect

While our study suggests a strong link between TAM-induced hepatotoxicity, gut microbial disturbances, and HDCA deficiency, several limitations should be noted. First, given that HDCA is far less abundant in humans than in rodents,^46^ future studies incorporating human bile acid profiling and clinical validation are warranted to enhance the translational relevance of our findings. Second, the causal role of *Lachnospiraceae*, particularly *the NK4A136 group*, in HDCA metabolism and TAM-induced liver injury remains insufficiently defined and requires future studies using germ-free or gnotobiotic models. Further clarification of microbial contributions and clinical validation are needed to better understand the relevance of these findings in human settings.

## Supplementary Material

Supplementary Material Supplementary_figures.docx

## Data Availability

The data analyzed in this study can be found in the National Library of Medicine at https://dataview.ncbi.nlm.nih.gov/object/PRJNA1271280?reviewer=n9es1os6l4q7bfh637n2hh9ls.

## Abbreviations

TAM: tamoxifen
BA: bile acid
DILI: drug-induced liver injury
GSH: reduced glutathione
GSSG: oxidized glutathione
ABX: a combination of antibiotics
PCoA: principal coordinates analysis
BSH: bile salt hydrolase
BaiN: 3-dehydro-bile acid delta 4,6-reductase
7α-HSDH: 7α-hydroxysteroid dehydrogenase
αMCA: alpha-muricholic acid
βMCA: beta-muricholic acid
ωMCA: omega-muricholic acid
CA: cholic acid
DCA: deoxycholic acid
LCA: lithocholic acid
CDCA: chenodeoxycholic acid
UDCA: ursodeoxycholic acid
HDCA: hyodeoxycholic acid
T-αMCA: tauro-alpha-muricholic acid
T-βMCA: tauro-beta-muricholic acid
T-ωMCA: tauro-omega-muricholic acid
TCA: tauro-cholic acid
TDCA: tauro-deoxycholic acid
TLCA: tauro-lithocholic acid
TCDCA: tauro-chenodeoxycholic acid
TUDCA: tauro-ursodeoxycholic acid
THDCA: tauro-hyodeoxycholic acid
FXR: farnesoid X receptor
FGF15: fibroblast growth factor 15
CYP7A1: cholesterol 7-alpha hydroxylase
CYP8B1: sterol 12-alpha-hydroxylase
CYP27A1: sterol 27-hydroxylase
CYP7B1: cytochrome P450 family 7 subfamily B member 1
OCA: obeticholic acid
CMC-Na: sodium carboxymethyl cellulose
TG: triglyceride
TC: total cholesterol
