# Supplementary material for: Tamoxifen induced hepatotoxicity via gut microbiota-mediated hyodeoxycholic acid depletion and Farnesoid X receptor signaling disruption
Source: Gut Microbes. 2026 Jan 2;18(1):2610077. doi: 10.1080/19490976.2025.2610077 (PMC12773634; doi:10.1080/19490976.2025.2610077)

**Supplementary Figure 1. TAM resulted in lower excretion of fecal bile acid and lower content of hepatic lipids.**

**(A)** Hepatic TC levels in mice undergoing 4-week (n= 10), 8-week (n= 5), or 16-week (n= 5) TAM treatment, respectively. **(B)** Hepatic TG levels. **(C)** The excretion of fecal bile acids. **(D)** Spearman correlation analysis between liver parameters and bile acids (TOP 5). Data were presented as the mean ± SEM. *, **, or *** indicated *p < 0.05*, *0.01*, or *0.001* representing the comparisons between control and TAM-treated groups, respectively. The symbol of ‘**ns**’ represented no significance among different comparisons.

**Supplementary Figure 2. Spearman correlation analysis was conducted between bacteria and bile acid profiles**

*, **, or *** in indicated *p < 0.05*, *0.01*, or *0.001* representing the comparisons between control and TAM-treated group, respectively.

**Supplementary Figure 3. Co-administration of GUS inhibitor did not mitigate TAM-induced liver injury.**

**(A and B)** Fecal GUS activity. **(C)** GUS inhibition models. **(D)** Liver weight, **(E)** liver index, and **(F)** serum ALP of Control-vehicle, Control-GUSi, TAM-vehicle, and TAM-GUSi (n= 8) mice with 4-week treatment, respectively. **(H)** HE staining of liver tissues. In Figure 2K, dashed box indicates the histological changes of hepatic vacuolation, while asterisks (*) represented the structure of hepatic central veins. **(G)** NAS score. Data were presented as the mean ± SEM. *** indicated *p <* *0.001* representing the comparisons between Control-vehicle and TAM-vehicle, respectively. ### indicated *p < 0.001* representing the comparisons between Control-GUSi and TAM-GUSi, respectively. The symbol of ‘**ns**’ represented no significance among different comparisons.

**Supplementary Figure 4. Antibiotic treatment significantly reduced fecal total bacterial load.**

* indicated *p <* *0.05* representing the comparisons between Control and ABX, respectively.

Supplementary Figure 1

**A**

**B**

**C**


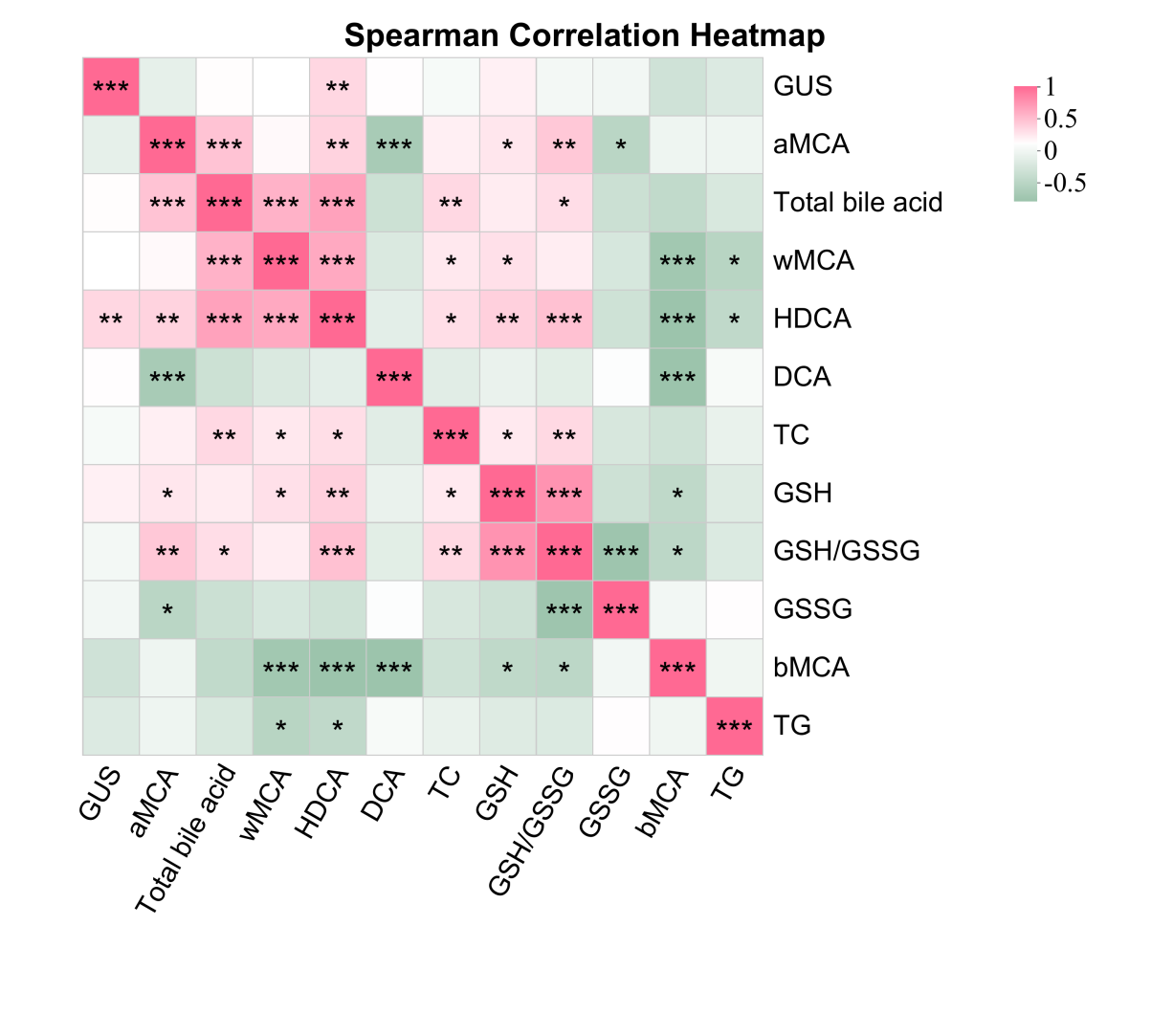


**D**


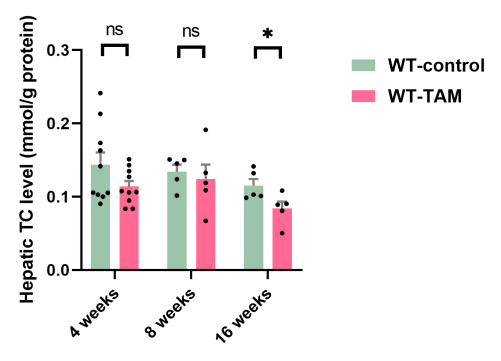

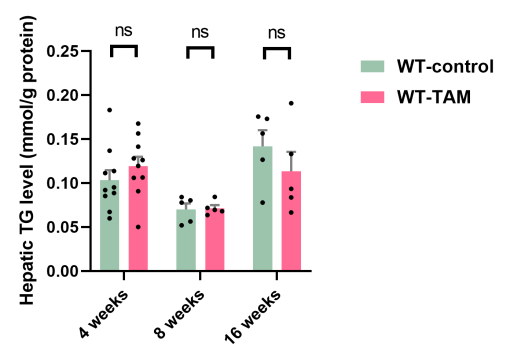

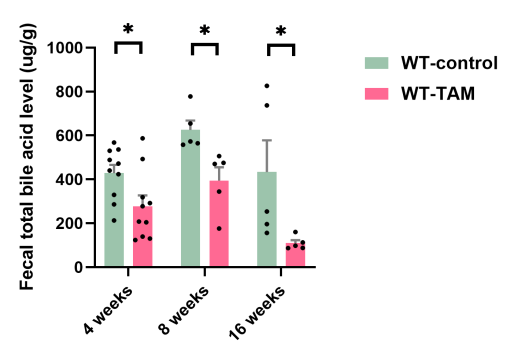


Supplementary Figure 2

Supplementary Figure 3


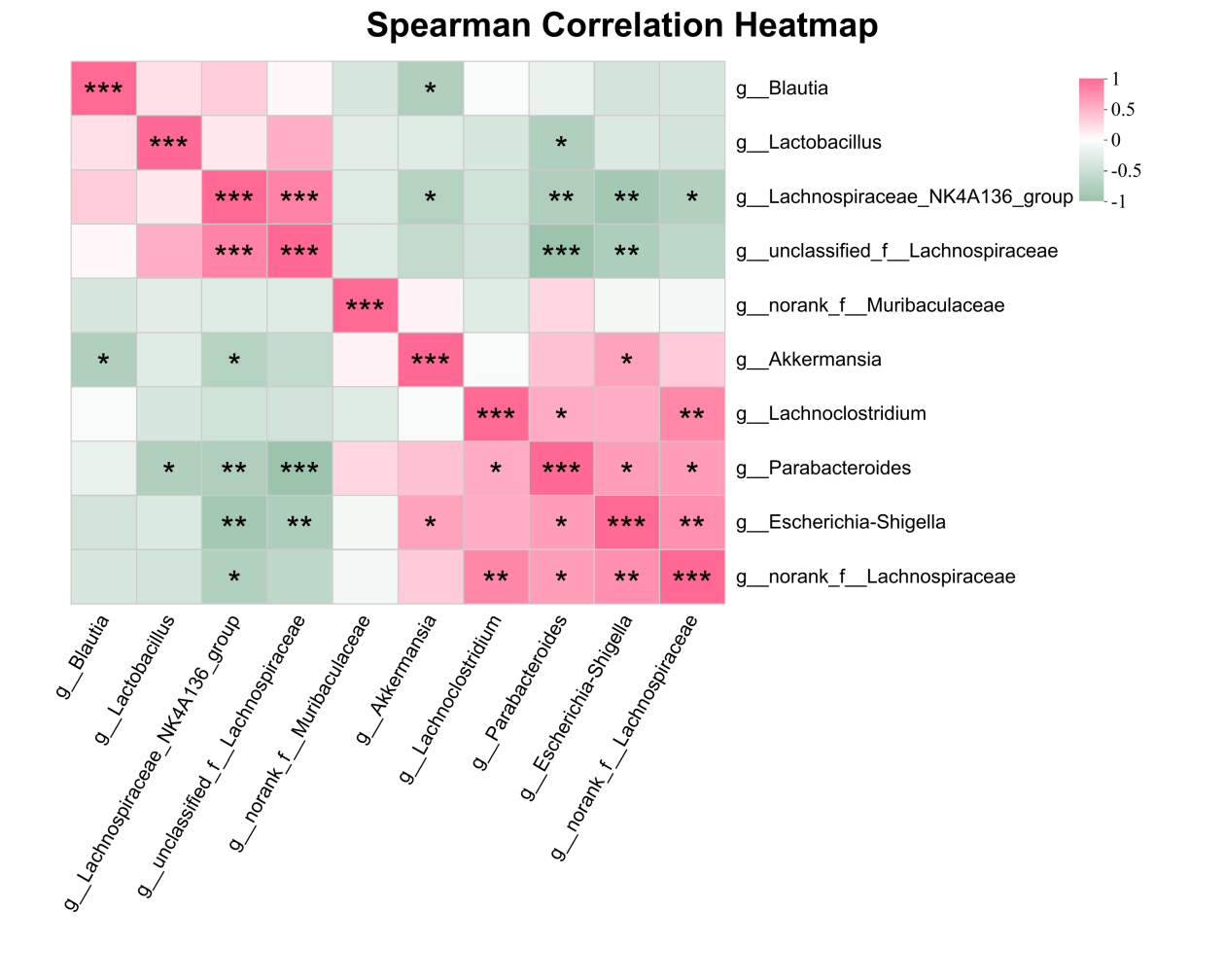

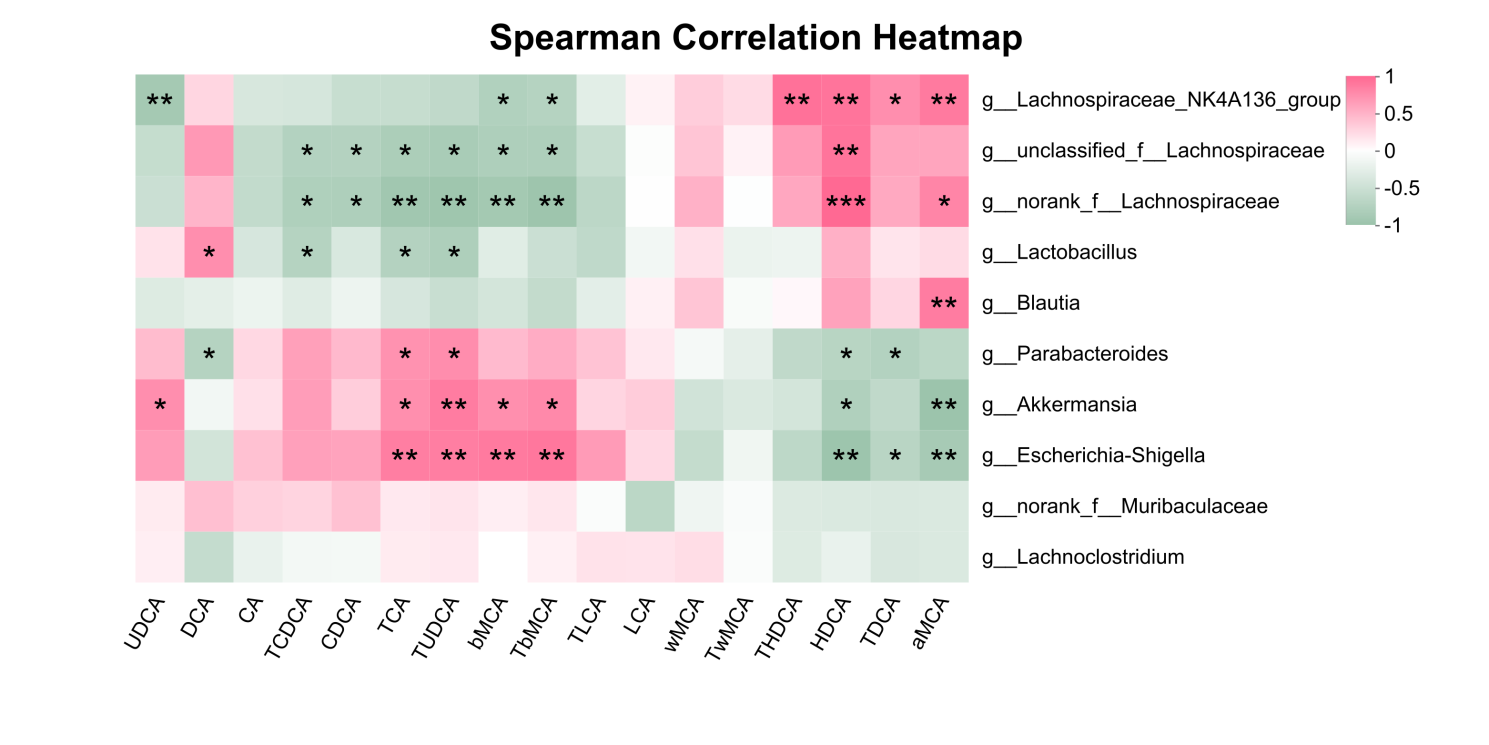


**A**

**B**

**Control-vehicle**

**Control-GUSi**

**TAM-vehicle**

**TAM-GUSi**


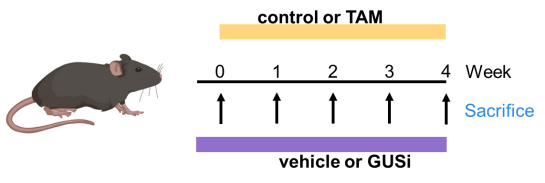


**A**

**B**

**C**

**D**

**E**

**F**

**G**

**H**


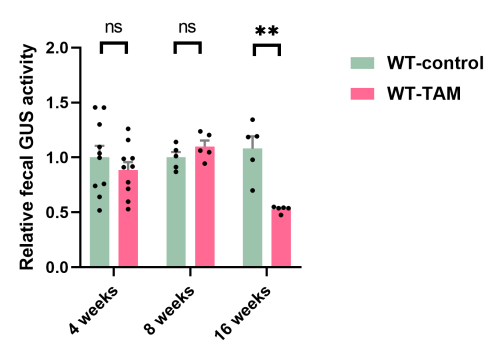

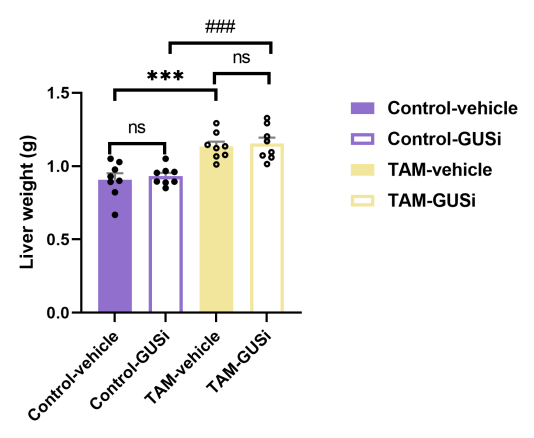

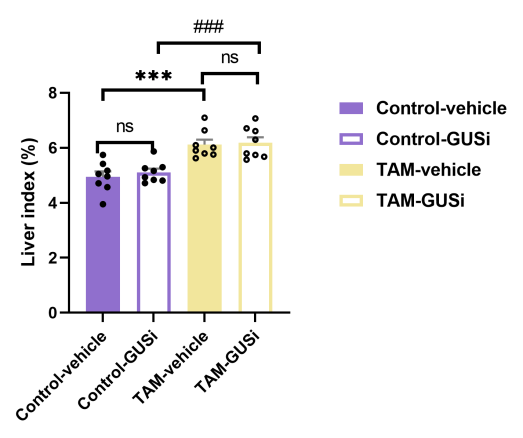

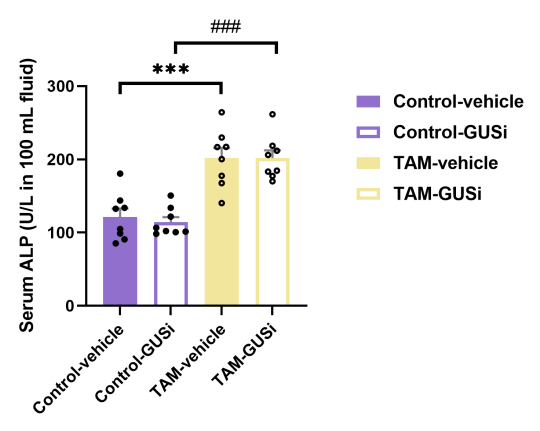

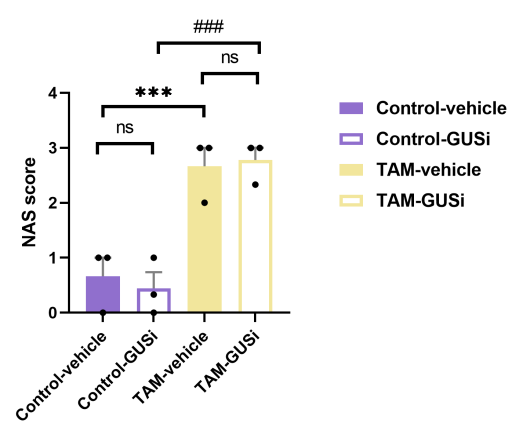

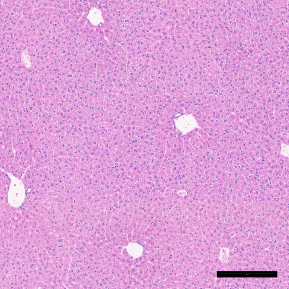

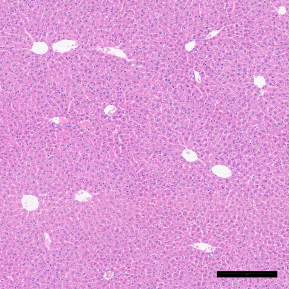

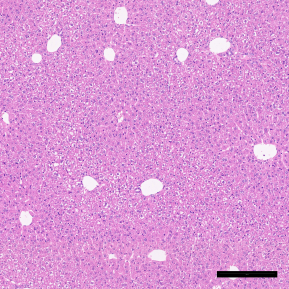

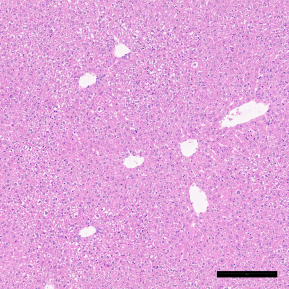


*****

*****

*****

*****

*****

*****

*****

*****

*****

*****

*****

*****

*****


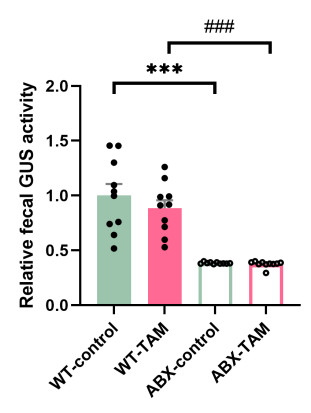

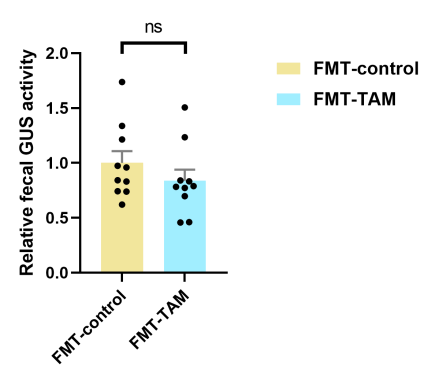


**I**

Supplementary Figure 4


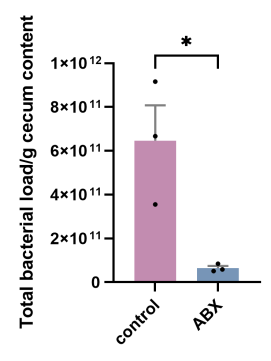

Supplement: Supplementary Material — Supplementary_figures.docx [file KGMI_A_2610077_SM6279.docx]
